# Anticonvulsant and Antioxidant Effects of Pitavastatin Against Pentylenetetrazol-Induced Kindling in Mice

**DOI:** 10.15171/apb.2017.035

**Published:** 2017-06-30

**Authors:** Nastaran Faghihi, Mohammad Taghi Mohammadi

**Affiliations:** ^1^Department of Physiology and Biophysics, Faculty of Medicine, Baqiyatallah University of Medical Sciences, Tehran, Iran.

**Keywords:** Seizure, Oxidative damage, Nitrosative damage, Pentylenetetrazole, Pitavastatin

## Abstract

***Purpose:*** The pleiotropic effects of statins (antioxidant and anti-inflammation) have been reported by previous studies. Therefore, we aimed to determine whether pitavastatin has protective effects against pentylenetetrazol (PTZ)-induced kindling in mice and also whether pitavastatin improves the brain antioxidant capacity and attenuates the oxidative injuries in kindled mice.

***Methods:*** Twenty-four mice were randomly divided into four groups (each group n=6); control, PTZ-kindling and PTZ-kindled rats treated with pitavastatin (1&4 mg/kg). PTZ kindling seizures were induced by repetitive intraperitoneal injections of PTZ (65 mg/kg) every 48 hours till day twenty-one. Animals received daily oral pitavastatin for twenty-one days. Latency, score and duration of the seizures were recorded. The activities of catalase (CAT) ad superoxide dismutase (SOD), and likewise the contents of malondialdehyde (MDA) and nitrate were assessed in the brains of all rats.

***Results:*** There was a progressive reduction in latency of the kindled rats in the next injections of PTZ. Pitavastatin reduced this value (latency) particularly at higher dose. Seizures duration and score also decreased in treatment groups. SOD and CAT activities significantly decreased in PTZ-kindling group by 62% and 64%, respectively, but pitavastatin did not significantly change the SOD and CAT activities. Brain MDA and nitrate significantly increased in PTZ-kindling group by 53% and 30%, respectively. Pitavastatin at higher dose significantly decreased the MDA and nitrate contents of PTZ-kindling rats by 45% and 32%, respectively.

***Conclusion:*** Our findings revealed that pitavastatin can improve the behavioral expression of the PTZ-kindling rats and attenuate the seizure-induced oxidative/nitrosative damage.

## Introduction

Epilepsy, the condition of recurrent unprovoked seizures, is one of the most common and serious brain disorders, which involves about 1% of individuals in worldwide.^1^ It's characterized by abnormal hypersynchronous paroxysmal cerebral discharges from a circumscribed region or large portions of the brain.^2^ Seizures frequently result from an imbalance of excitation and inhibition due to a failure of inhibitory neurotransmission.^3,4^ The evidence linking epilepsy with dysfunction of GABAergic inhibition is substantial and GABA_A_ receptor is a major target of antiepileptic drugs (AED).^5^ Using the drugs that impacts the ion channels of cell membranes such as GABA receptors is useful to control the seizures in approximately 70 percent of epileptic patients.^1,5^ However, for remaining patients that have intractable seizures (30 percent) new antiepileptic drugs have slight effects.^6^ Therefore, new attitudes are needed to treat these patients.

Several animal models and genetic studies have confirmed the connection between oxidative stress and seizure.^7,8^ Previously, studies have proven that status epilepticus alter the redox potential and reduce the level of ATP, which can result in failure of brain energy production and supply.^9,10^ Recently, Waldbaum *et al.* reported acute injuries induced by reactive oxygen species (ROS) contribute to formation of chronic epilepsy.^11^ Likewise, ROS accumulation, mitochondrial dysfunction, lipid peroxidation and brain edema have been observed following prolonged epileptic seizures.^8,9^ Lipid peroxidation in patients with seizure is significantly higher compared to normal people.^7,12^ Liang and Patel have also verified oxidative damage to susceptible targets (protein, lipids, and DNA) caused by seizure.^13,14^ Therefore, reducing oxidative damage may ameliorate tissue damage and occurrence of seizure.

Statins are competitive inhibitors of HMG-CoA (3-hydroxyl-3-methyl-glutaryl coenzyme A) reductase, which inhibit the rate-limiting enzyme of the mevalonate pathway for cholesterol biosynthesis. Pleiotropic effects of statins are other important advantages of using these drugs that are including the improving endothelial function, anti-oxidative stress, neuroprotection, anti-inflammation, and anti-neurotoxicity.^15,16^ Recently, studies have suggested the anticonvulsive effects of statins. It has been shown that atorvastatin efficiently reduces the kainic acid-induced seizure activities, hippocampal neuronal death and pro-inflammatory genes expression.^17^ Also, oral atorvastatin treatment has increased the latency to pentylenetetrazol (PTZ)-induced generalized seizures.^18^ The findings of Rangel *et al.* indicated that lovastatin exerted a neuroprotective role in attenuation of brain damage after pilocarpine-induced status epilepticus.^19^ Moreover, Xie *et al.* reported simvastatin suppressed cytokines expression and reactive astrocytosis and also decreased the frequency of discharges of epileptic brain.^20^ These observations suggest a potential for use of statins in modulation of seizure behaviors. However, there is no study to show the effects of pitavastatin on seizure or epilepsy.

In the present study, we aimed to examine the protective effects of pitavastatin in PTZ kindling model of seizure in mice. Furthermore, the purpose of our study was to determine whether pitavastatin improve the antioxidant capacity of cerebral tissues and reduce oxidative/nitrosative damages during seizure.

## Materials and Methods

### Animals 

The protocols of all experiments were approved by institutional Animal Ethics Committee of Baqiyatallah University of Medical Sciences (Tehran, Iran). Adult male NMRI mice (7-8 weeks old, 23±7 gr) were obtained from Animal House Facility of the University of Baqiyatallah Medical Sciences. The mice were kept in standard cages in a room with controlled light period (07.00 a.m. to 19.00 p.m.), humidity (40-60%), temperature (22-24°C), and also rats were given *ad libitum* access to rat chow and water.

### Chemicals

Pitavastatin was purchased from Kowa Pharmaceutical Co Ltd, Tokyo, Japan. Pentylenetetrazole (PTZ) was obtained from Sigma, Germany. PTZ was dissolved in physiological saline solution for intraperitoneal (*i.p.*) injection. Pitavastatin was dissolved in sterile distilled water and administrated orally (by gavage) in treatment groups, while untreated mice received sterile distil water in same volume.

### Experimental design

Four groups of mice (N=24) were used in the present study (for each group; n= 6). The mice were randomly divided into Control, PTZ (PTZ kindling model of seizure) and two PTZ groups treated with pitavastatin (1 and 4 mg/kg). Seizures were induced in mice by the repeated intraperitoneal injections of PTZ (65 mg/kg) every 48 hours.^21,22^ PTZ injection was continued till day 21. Treated mice received orally pitavastatin 5 days before induction of seizures till day 21 at doses of 1 and 4 mg/kg/day. Moreover, untreated mice received saline solution (*i.p.*) like treated mice in same volume.

### Determination of score, latency and duration of seizures

Immediately after PTZ injection in PTZ kindling model of seizure, the animals were transferred to a round open filed and seizure score was monitored. Latency or the time elapsed between injection of a convulsive dose of PTZ (65 mg/kg) and beginning of seizure was recorded. The seizures scores were classified as followed stage 0: no response, stage 1: eat and facial twitching, stage 2: myoclonic body jerks, stage 3: forelimb clonus, rearing, stage 4: clonic convulsions, turn onto the side and stage 5: generalized clonic convulsions, turn onto the back. Finally, the length of seizures was recorded as the seizure duration.^23^

### Preparation of brain tissues 

The brain tissues of mice were rapidly removed under deep anesthesia to determine the brain contents of nitrate and malondialdehyde (MDA) as well as to assay the activities of catalase (CAT) and superoxide dismutase (SOD) enzymes. The brains were quickly washed in ice-cold phosphate buffer saline (PBS) and immediately frozen by liquid nitrogen. The frozen tissues were rapidly weighed and homogenized in ice-cold PBS (1:10). After centrifuging (14000×g for 15 min at 4°C), the supernatants were used to determine the brain contents of nitrate, MDA and protein as well as the enzymes activities of SOD and CAT. 

### Determination of superoxide dismutase (SOD) activity

According to the ability of SOD to inhibit the reduction of nitroblue tetrazolium (NBT; Sigma, Germany) by superoxide, the activity of SOD was determined. “For assay, 0.067 M potassium phosphate buffer, pH 7.8 was added to 0.1 M EDTA containing 0.3 mM sodium cyanide, 1.5 mM NBT and 0.1 mL of sample. Then, 0.12 mM riboflavin (Sigma, Germany) was added to each sample to initiate the reaction and was incubated for 12 min. The absorbance of samples was read on a Genesys 10 UV spectrophotometer (CECIL-2501, England) at 560 nm for 5 min. The amount of enzyme required to produce 50% inhibition was taken as 1 U and results were expressed as U/mg protein”.^24^

### Determination of catalase (CAT) activity

To determine the brain activity of catalase (CAT), “reaction mixture, containing 0.85 mL potassium phosphate buffer 50 mM (pH; 7.0) and 0.1 mL tissue homogenate, was incubated at room temperature for 10 min. Reaction was initiated by addition of 0.05 mL H_2_O_2_ (30 mM prepared in potassium phosphate buffer 50 mM, pH 7.0) and the decrease in absorbance was recorded for 3 min at 240 nm. Specific activity is expressed as 1µmol H_2_O_2_ decomposed U/mg protein”.^25^

### Assessment of malondialdehyde (MDA) 

The brain content of MDA (end product of lipid peroxidation) was measured as the index of oxidative stress. First, “0.5 mL of tissue homogenate was added to 1.5 mL of 10% TCA (trichloroacetic acid; Sigma, Germany), vortexed and incubated for 10 min at room temperature. 1.5 mL of supernatant and 2 mL of thiobarbituric acid (0.67%; Sigma, Germany) were added and placed in a boiling water bath in sealed tubes for 30 min. The samples were allowed to cool at room temperature. 1.25 mL of n-butanol (Sigma, Germany) was added, vortexed and centrifuged at 2000 g for 5 min. The resulting supernatant was removed and measured at 532 nm on a spectrophotometer. MDA concentrations were determined by using 1,1,3,3-tetraethoxypropane as the standard. Finally, the MDA concentration was expressed as µg/mg protein”.^26^

### Assessment of nitrate content

The nitrate content (the main metabolite of nitric oxide, NO) of brain, as the index of NO, was determined by the colorimetric reaction of Griess reagent. “0.1 mL of homogenate solution was deproteinized by adding 0.2 mL of zinc sulfate solution and centrifuged for 20 min at 4000 g and 4 °C to separate supernatant for nitrate assay. 0.1 mL of supernatant (as sample) or pure water (as blank) or sodium nitrite (as standard; Sigma, Germany) was mixed with 0.1 mL vanadium III chloride (Sigma, Germany). 0.05 mL sulfanilamide (0.01 %; Sigma, Germany) and 0.05 mL N-[1-naphthyl] ethylene-diamin-dihydrochloride (NED, 0.01 %; Sigma, Germany) were incubated for 30 min in dark place at 37 °C. Thereafter, the absorbance of solution was determined at wave length of 540 nm. Nitrate concentration was estimated from a standard curve generated from absorbance of each sodium nitrate solution. Finally, the nitrate content was expressed as µg/mg protein”.^27^

### Assessment of protein concentration

Based on Bradford method, protein levels of the brain tissues were determined.^28^ To calculate the protein concentration, bovine serum albumin (BSA; Sigma, Germany) was used as a standard.

### Statistical analysis 

SPSS (V.21) was used for statistical analysis. All data were presented as mean±SEM. One-way variance (ANOVA) and Tukey's Post-Hoc test was used to compare the data between control and PTZ (treated and untreated kindled mice) groups. Also, repeated measure was used to analyze the data of different times for latency, duration and score of seizures in each group. All states, *p<0.05* was considered as significant difference.

## Results

### Effect of pitavastatin on latency time

**Figure 1:**
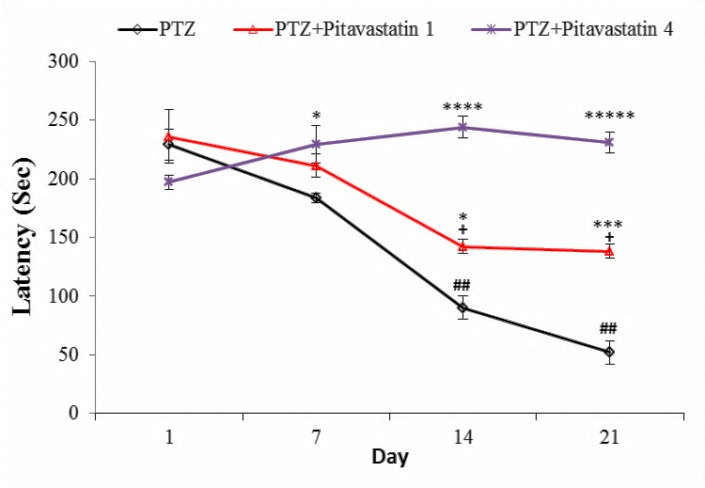
The latency of untreatment kindled group (PTZ) and kindled mice treated with 1 mg/kg (PTZ+Pitavastatin 1) and 4 mg/kg (PTZ+Pitavastatin 4) pitavastatin. All values are presented as Mean±SEM. *P<0.05, ***P<0.001, ****P<0.0001 and *****P<0.00001 as significant difference compared to PTZ group for own day ^##^P<0.01 and ^###^P<0.001 as significant difference compared to PTZ group at day one ^+^P<0.05 as significant difference compared to kindled mice treated with 1 mg/kg day one

Latency or the time elapsed between injection of a convulsive dose of PTZ (65 mg/kg) and beginning of seizure is presented in Figure 1. The latency of PTZ group (untreated kindled mice) was 229±13 Sec at day one that decreased progressively in the next injections of PTZ. So the reduction of latency at days of 14 (90±10 Sec) and 21 (52±10 Sec) statistically differed compared with day one. Likewise, the progressive reduction of latency was observed in kindled mice treated with 1 mg/kg pitavastatin but this reduction was mild. The value of latency for this group was 142±6 and 138±6 Sec at days 14 and 21, respectively, that statistically differed compared to PTZ group. However, pitavastatin at dose of 4 mg/kg prevented the progressive reduction of latency in kindled mice. This value was 229±16, 244±9 and 231±9 Sec at days 7, 14 and 21, respectively, which were near to the latency of day one (197±6 Sec).

### Effect of pitavastatin on seizure length 

**Figure 2:**
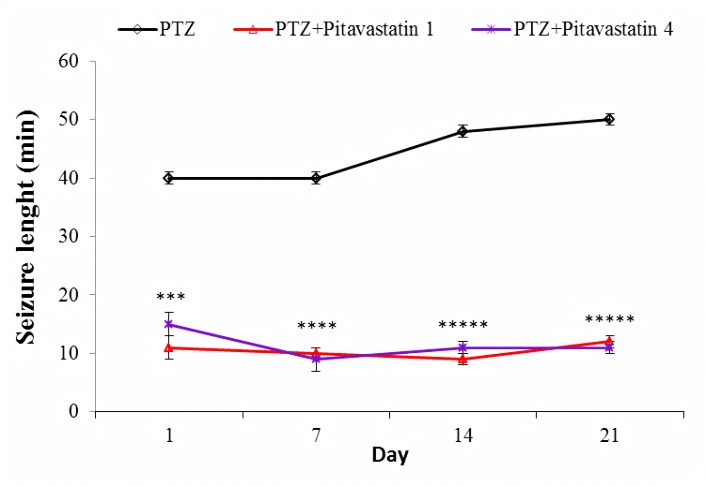
Seizure length of untreatment kindled group (PTZ) and kindled mice treated with 1 mg/kg (PTZ+Pitavastatin 1) and 4 mg/kg (PTZ+Pitavastatin 4) pitavastatin. All values are presented as Mean±SEM. ***P<0.001, ****P<0.0001 and *****P<0.00001 as significant difference compared to PTZ group for own day

Figure 2 is showing the seizure length of kindled mice during the test. The seizure length of PTZ group (untreated kindled mice) was 40±1 at day one. This value did not significantly alter in the next injections of PTZ. Administration of pitavastatin considerably decreased the values of seizure length in both treatment kindled groups during the test. This reduction was same in both kindled groups treated with pitavastatin and there was no significant difference between them.

### Effect of pitavastatin on seizure score 

**Figure 3:**
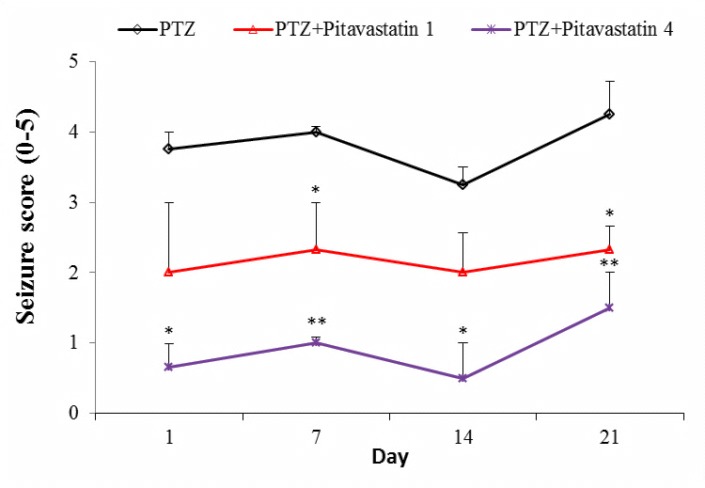
Score of seizure (0-5) in untreatment kindled group (PTZ) and kindled mice treated with 1 mg/kg (PTZ+Pitavastatin 1) and 4 mg/kg (PTZ+Pitavastatin 4) pitavastatin. All values are presented as Mean±SEM. *P<0.05 and **P<0.01as significant difference compared to PTZ group for own day

Seizure score, the severity of seizure in kindled mice, is shown in Figure 3. The control mice did not show any seizure signs during the test. Mean value of seizure score in untreatment kindled mice (PTZ group) was 3.75±0.25 at day one. The value of seizure score did not change in the next injections of PTZ in PTZ group. Pitavastatin administration at dose of 1 mg/kg decreased the values of seizure score in the next injections. The values of days 7 and 14 significantly differed compared to PTZ group. Likewise, pitavastatin at dose of 4 mg/kg reduced the values of seizure score in the next injections of PTZ more than 1 mg/kg.

### Effect of pitavastatin on SOD activity

**Figure 4:**
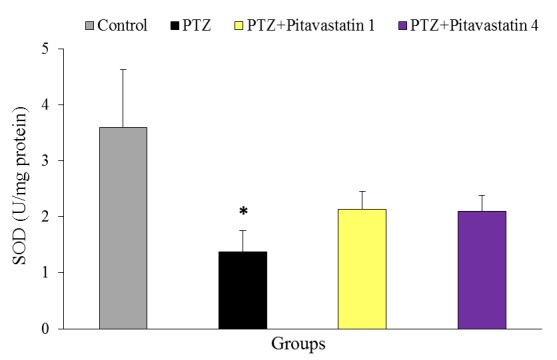
Brain activity of superoxide dismutase, SOD, (U/mg protein) at the end of experiment in groups of control, untreatment kindled group (PTZ) and kindled mice treated with 1 mg/kg (PTZ+Pitavastatin 1) and 4 mg/kg (PTZ+Pitavastatin 4) pitavastatin. All values are presented as Mean±SEM. * As significant difference compared to control (P<0.05)

The activity of brain SOD is presented in Figure 4 at termination of the experiment. The value of SOD significantly reduced in untreatment kindled mice (1.37±0.39 U/mg protein) in comparison with control mice (3.60±1.03U/mg protein). Pitavastatin at doses of 1 and 4 mg/kg did not significantly decrease the SOD activity of brain in treatment kindled mice compared to untreatment kindled mice. The value of SOD was 2.13±0.32 and 2.10±0.28 U/mg protein for treatment kindled groups at doses of 1 and 4 mg/kg pitavastatin, respectively.

### Effect of pitavastatin on CAT activity

**Figure 5:**
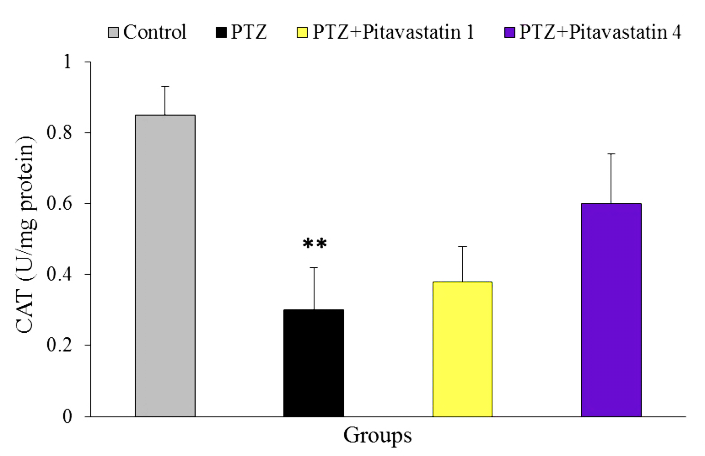
Brain activity of catalase (CAT, U/mg protein) at the end of experiment in groups of control, untreatment kindled group (PTZ) and kindled mice treated with 1 mg/kg (PTZ+Pitavastatin 1) and 4 mg/kg (PTZ+Pitavastatin 4) pitavastatin. All values are presented as Mean±SEM. ** as significant difference compared to control (P<0.05)

Figure 5 shows the activity of brain CAT at termination of the experiment. This value significantly decreased in untreatment kindled mice (0.30±0.12 U/mg protein) in comparison with control mice (0.85±0.08 U/mg protein). Pitavastatin at doses of 1 and 4 mg/kg did not significantly decrease the CAT activity of brain in treatment kindled mice compared to untreatment kindled mice. This value was 0.38±0.10 and 0.60±0.14 U/mg protein for kindled groups treated with 1 and 4 mg/kg pitavastatin, respectively.

### Effect of pitavastatin on MDA content

**Figure 6:**
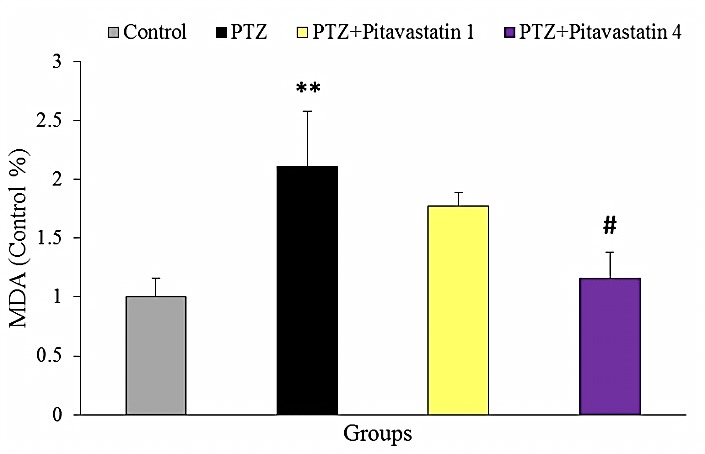
Relative content of brain MDA (Control %) at the end of experiment in groups of control, untreatment kindled group (PTZ) and kindled mice treated with 1 mg/kg (PTZ+Pitavastatin 1) and 4 mg/kg (PTZ+Pitavastatin 4) pitavastatin. The graph has been represented as the relative changes calculated by normalization to the data of control group. All values are presented as Mean±SEM. ** as significant difference compared to control (P<0.01) ^#^ as significant difference compared to PTZ group (P<0.05)

The relative concentration of brain MDA (Control %), as an index of lipid peroxidation, was shown in Figure 6 at termination of the experiment. The value of MDA significantly increased in untreatment kindled mice (PTZ group) by 53% compared to the control mice. Pitavastatin at dose of 1 mg/kg did not noticeably decrease the value of brain MDA of treatment kindled mice. However, pitavastatin at dose of 4 mg/kg significantly decreased the value of MDA in treatment kindled mice by 45% compared to untreated PTZ group.

### Effect of pitavastatin on nitrate content

**Figure 7:**
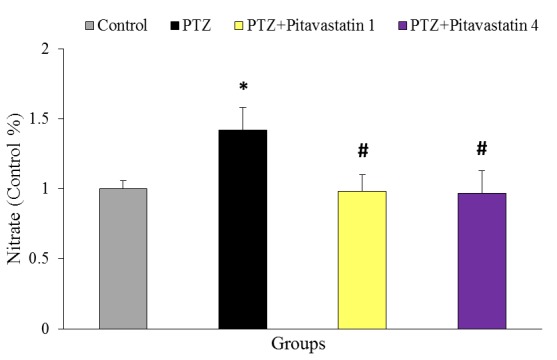
Relative content of brain nitrate (Control %) at the end of experiment in groups of control, untreatment kindled group (PTZ) and kindled mice treated with 1 mg/kg (PTZ+Pitavastatin 1) and 4 mg/kg (PTZ+Pitavastatin 4) pitavastatin. The graph has been represented as the relative changes calculated by normalization to the data of control group. All values are presented as Mean±SEM. * as significant difference compared to control (P<0.05) ^#^ as significant difference compared to PTZ group (P<0.05)

Figure 7 is showing the relative content of brain nitrate (Control %) at the end of experiment. The value of nitrate, as the index of NO production, significantly increased in untreatment kindled mice (PTZ group) by 30% compared to the control mice. Pitavastatin significantly decreased the value of brain nitrate in both treatment kindled groups (1 and 4 mg/kg) compared to PTZ group by 31% and 32%, respectively.

## Discussion

Recently, many studies have reported the anticonvulsive effects of several statins such as atorvastatin and rosuvastatin.^15,19,20^ In the present study, we showed that pitavastatin has anticonvulsive effects by improving the seizure behaviors such as increment of latency, reduction of durations and scores in PTZ kindling model of seizure. Treatment with pitavastatin did not noticeably enhance the activities of antioxidant enzymes (SOD and CAT) during seizures but pitavastatin considerably decreased the brain oxidative or nitrosative damage of kindled mice (reduction of brain MDA and nitrate). Likewise, pitavastatin in higher dose (4 mg/kg) was more effective compared to lower dose (1 mg/kg). Since free radicals play an important role in pathophysiology of seizure,^9,11^ it is suggested that anticonvulsive effects of pitavastatin correlate with antioxidant effects of this compound.

The PTZ kindling model has been developed to study of the seizure in animal models. It has been identified that PTZ is a non-competitive antagonist of GABA_A_ receptor and has convulsive effects after repetitive or single administration.^21^ Our findings indicated that the injections of PTZ (65 mg/kg) were able to induced seizure in mice. The data of score as well as duration and latency confirmed the seizures in our study. These results are in agreement with the findings of other documented studies.^21,29^ Several reports have also indicated that free radicals play a crucial role in pathogenesis of seizure behaviors of the PTZ-kindled animals.^7,8,12^ The findings of current study are indicating that the contents of oxygen and nitrogen free radicals (brain MDA and nitrate) were considerably enhanced following kindling. These free radicals lead to cellular dysfunction by attacking the polyunsaturated sites of the biological membranes resulting into increased lipid peroxidation.^13,14^ Also, it must be mentioned that brain is highly susceptible to oxidative stress because of high demand for oxygen and abundant polyunsaturated fatty acids. Our findings are indicating a significant reduction of the brain antioxidant enzymes following kindling because there was a considerable reduction in the activities of SOD and CAT. SOD is the main antioxidant enzyme of brain and CAT is an enzyme responsible for detoxification of H_2_O_2_ formed by the action of SOD.^30,31^ Also, it must be mentioned that the ability of the antioxidant defense system of brain against ROS is feeble compared to other tissues, therefore brain cells are susceptible to more damages by free radicals.^31^ Finally, it is suggested that weakening of brain antioxidant system and oxidative stress may play a crucial role in pathophysiology of seizure.

Many clinical and experimental findings have demonstrated the beneficial effects of statins on neurological diseases such as stroke and Alzheimer’s disease.^16,30,32^ Although there are limited studies indicating the anticonvulsive effects of statins,^18-20^ there is no study about the anticonvulsive effects of pitavastatin. The findings of current study indicated that pitavastatin was able to increase the seizures latency and reduce the scores and durations of seizures. There was a progressive reduction in latency of PTZ-induced seizure at the next injections of PTZ, whereas pitavastatin particularly in higher dose (4 mg/kg) prevented this progressive reduction. These findings are indicating that pitavastatin like other statins has anticonvulsive effects. Pleiotropic effects of pitavastatin, including anti-oxidative stress and anti-inflammation properties as well as anti-apoptotic effects, have been proven by several recent studies.^16,33-35^ It seems that these effects of pitavastatin be important in anticonvulsive effects of this compound like other statins. Reported, treatment with atorvastatin for seven days increases the latency of PTZ-induced seizure in mice.^18^ Atorvastatin pretreatment has also reduced the hippocampal neural death and seizure activity of kainic acid-induced seizure.^17^ Moreover, Seker *et al.* reported that rosuvastatin reduces epileptic form activity which was associated with improved blood-brain barrier integrity.^15^

Oxidative stress and mitochondrial dysfunction have been determined as a key factor in pathogenesis of many neurodegenerative diseases as well as epilepsy and seizure.^9,31^ In the current study, pitavastatin considerably decreased the content of MDA, as a main index of ROS, particularly in higher dose (4 mg/kg). It has been reported that increment of free radicals and mitochondrial dysfunction are associated to initiation and progression of epilepsy.^8,12^ Based on previous studies, ROS may lead to Ca^2+^-dependent depolarization of mitochondrial membrane potential and ATP reduction, which can promote the processes of status epilepticus.^10^ Since the antioxidant effects of pitavastatin has been confirmed in many pathological states of brain,^33,34^ it is concluded that pitavastatin has anticonvulsive and antiepileptic properties through its power antioxidant effects. In the present study pitavastatin greatly decreased the content of nitrate, as a main index of nitric oxide (NO). NO has been implicated in the promoting of oxygen toxicity in brain. NO promotes the progression of epilepsy by induction of neural loss and reactive glial proliferation.^36^ Moreover, high level of NO can react with superoxide anions resulting in peroxynitrite formation, which is a power inducer of cell death.^37^ Therefore, it is suggested that pitavastatin modulates PTZ-induced seizure and improves seizure behaviors possibly by inhibition of the toxic effects of NO. However, more studies need to verify how pitavastatin reduce the brain NO in PTZ-induced seizure.

Our findings confirmed that the brain antioxidant enzymes such as SOD and CAT inactivate during seizure. SOD is an important antioxidant enzyme that quenches the superoxide anions in mitochondrial matrix.^10^ Reported, reduction in SOD level during epileptogenesis may promote the neural death and brain damage.^38^ Likewise, CAT is another antioxidant enzyme of brain, which can detoxify the H_2_O_2_ to form water and molecular oxygen.^31^ Freitas *et al.* reported that CAT is an important antioxidant enzyme that plays protective role in hippocampus against status epilepticus.^39^ In the current study, pitavastatin particularly in higher dose could enhance the activity of these enzymes in treated epileptic mice, whereas, the values of these enzymes (SOD and CAT) did not statistically differ compared to untreatment kindled mice. It is suggested that the intensity of seizures was great. Reported, other statins were able to increase the activities of antioxidant enzymes of brain such as SOD and CAT in many pathological states.^16,30,33^ Therefore, other studies need to clarify the direct effects of pitavastatin on activities of the brain antioxidant enzymes during seizure.

## Conclusion

In conclusion, the findings of present study suggest that chronic administration of pitavastatin is able to modulate the behaviors of seizures, which may be related to inhibition of the seizure-induced oxidative/nitrosative damage. It is concluded that treatment with pitavastatin may be helpful to prevent the seizure and seizure-induced brain damage beside the other anticonvulsant drugs.

## Acknowledgments

The authors are cordially appreciating Mr. Mark Azad for providing the drugs and Mrs. Nahid Sarahian for helping in surgical protocols.

## Ethical Issues

Not applicable.

## Conflict of Interest

The authors declare no conflict of interests.
